# The Student-Authored Biomedical Publications at Alfaisal University, Saudi Arabia: a 6-year descriptive analysis

**DOI:** 10.1186/s40064-015-1551-0

**Published:** 2015-12-02

**Authors:** Asma Alnajjar, Tehreem A. Khan, Syeda Mina, Khaled Alkattan, Ahmed Abu-Zaid

**Affiliations:** College of Medicine, Alfaisal University, P.O. Box 50927, Riyadh, 11533 Saudi Arabia

**Keywords:** Medical students, Publications, Research, PubMed, Alfaisal University, Saudi Arabia

## Abstract

There are limited numbers of studies which comprehensively explored the research publications authored by medical students. To descriptively analyze the student-authored research publications originating from Alfaisal University—College of Medicine (Riyadh, Saudi Arabia) over a 6-year period. All student-authored research publications were retrieved from PubMed^®^ and the College’s publication database. Study inclusion criteria included: (1) at least one medical student author, (2) *published* and/or *accepted in*-*press* PubMed-indexed article from 10 September 2008 to 31 December 2014. Data was transferred to Microsoft Excel Software for descriptive statistical analysis of variable parameters. Seventy-three (n = 73) articles met the study inclusion criteria. They were published by 170 students; the majority were males (79.4 %) and clerkship students (65.9 %). There was a markedly steady increase in number of yearly publications from 1 publication in 2009 to 35 publications by the end of 2014. Fifty (68.5 %), twenty-nine (39.7 %) and thirty-seven (50.7 %) students were first, second and corresponding authors, respectively. The most frequent research areas were clinical science (43.8 %), basic science (23.3 %) and medical education (21.9 %). The most frequent research types were case reports (41.1 %), research articles (32.9 %) and correspondence letters (15.1 %). Fifty-seven (78.1 %) and sixteen (21.9 %) publications took place in local and abroad institutes, respectively. Most publications (71.2 %) had impact factors below 2. The mean ± SD of articles’ impact factors and citations were 3.9 ± 9.9 and 1.9 ± 4.1, respectively. Students demonstrated positive attitudes towards publishing and significantly contributed to the institution’s pool of research publications.

## Background

In the twenty-first century, research is the primary backbone of advancing biomedical sciences and improving healthcare (Al-Halabi et al. 2014). The call for formal integration of scholarly research training into undergraduate medical curricula is becoming increasingly underscored (Abu-Zaid and Alkattan 2013; Boyer Commission on Educating Undergraduates in the Research University 1998; World Federation for Medical Education 2015). At present, proficiency in scientific research theoretically (knowledge-wise) and practically (skills-wise), regardless of healthcare specialty, is rapidly emerging as a central competency for all the 21st-century medical generations (Abu-Zaid 2014). In line with the above-mentioned emerging trend, the Saudi Meds (a national competence specification for Saudi medical graduates) was developed through a consensus of several medical colleges in Saudi Arabia. One of its seven domains is “the doctor and research domain”. This domain dictates that medical students should: (a) get introduced to undergraduate research activities early in medical education, (b) foster genuine understanding of the significance of scientific research in the medical arena, and (c) conduct various student-led and faculty-supervised undergraduate scholarly activities (Zaini 2011).

Research is incomplete and lacks substantial rewards without knowledge dissemination. Generally speaking, knowledge dissemination can take place through: (1) publication in professional peer-reviewed journals, or (2) presentation at scientific meetings. While the latter is associated with dynamic knowledge dissemination, the former embraces higher values when assessing applicants for postgraduate opportunities (Green et al. 2009; Marwan and Ayed 2013; Seymour et al. 2004; Sinha et al. 2010).

Medical students can contribute significantly to an institution’s pool of research publications (Aslam et al. 2005). In fact, research publications by medical students are an age-old and rapidly increasing phenomenon (Wickramasinghe et al. 2013).

Founded on 10 September 2008, Alfaisal University—College of Medicine, Riyadh, Saudi Arabia, is a private, non-profit, student-centered educational institute. Research does not represent a “formal” curricular component, and research thesis is not pre-requisite for graduation and earning the Bachelor of Medicine, Bachelor of Surgery (MBBS) degree. However, scientific research activities are greatly encouraged and pursued at this young institution. These research activities are largely facilitated by the College’s Undergraduate Research Committee (Alamodi et al. 2014). Medical Students are always motivated and facilitated to conduct both, local and international student-run and faculty-mentored scholarly research projects which often culminate in peer-reviewed publications. Faculty staff is also incentivized to mentor undergraduate research projects. As a consequence, despite its young age, Alfaisal University—College of Medicine has an ever-growing research publication database.

There are limited numbers of studies which comprehensively explored the research publications authored by medical students (Griffin and Hindocha 2011). To the best of our knowledge, no previous studies have been conducted on investigating the student-authored research publications from our medical college, or other local medical colleges in Saudi Arabia.

The objective of this study is to descriptively analyze and explore the student-authored research publications originating from Alfaisal University—College of Medicine over a period of 6 years.

## Methods

The study took place at Alfaisal University—College of Medicine (Riyadh, Saudi Arabia) and was approved by the respective Institutional Review Board (IRB).

All publications originating from Alfaisal University—College of Medicine were retrospectively retrieved from PubMed^®^ as well as the up-to-date research database managed by the College’s Undergraduate Research Committee.

The study inclusion criteria for the student-authored research publications included: (1) at least one full-time medical student author, and (2) *published* and/or *accepted in*-*press* PubMed-indexed articles from 10 September 2008 to 31 December 2014.

Published manuscripts were verified by checking the respective abstracts on PubMed^®^. On the other hand, accepted in-press manuscripts were verified upon receiving official evidence (email/document) from the medical student author and/or journal editorial office. The combined database was scanned for duplicate publications and omitted accordingly.

The study’s major exclusion criteria for student-authored publications included: (1) publications taking place outside the pre-determined period of 10 September 2008 to 31 December 2014, (2) publications authored solely by in-house faculty, (3) *submitted* manuscripts that are undergoing peer-review and pending editorial decisions, (4) publications in non-PubMed-indexed journals, and (5) publications other than journal manuscripts such as book chapters.

For every eligible student-authored research publication, the following details were recorded and analyzed: number of male/female authors, number and gender of students as first/second/corresponding authors, research area, research type, publication year, journal’s impact factor, number of citations per article, academic year of student authors at the time of publication, and origin of research institute.

Research areas included: basic science, clinical science, medical education and epidemiology/public health. Research type (determined as per the journals’ categorization) included: research article, short communication, review article, case report, letter to editor (correspondence) and others. Journals’ impact factors were calculated as per Journal Citation Reports^®^ Thomson Reuters for 2013/2014. Number of article citations was checked using Google Scholar^®^. Origin of research institute included: local (Alfaisal University—College of Medicine and King Faisal Specialist Hospital and Research Center, Riyadh, Saudi Arabia) and aboard (United States of America and Canada).

Data was transferred to Microsoft Excel 2010 Software (Microsoft™, Redmond, WA, USA) for descriptive statistical analyses such as: numbers, percentages, and means ± standard deviations. Data was simplified into tables and figures—where deemed appropriate.

## Results

**Fig. 1:**
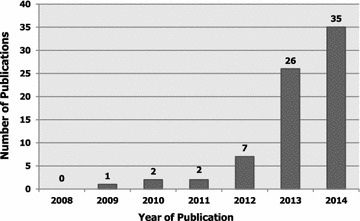
Year-wise distribution of the student-authored research publications over a 6-year period from 2008 to 2014 (n = 73)

Seventy-three published and/or accepted-in-press manuscripts met the study inclusion criteria for student-authored research publications. Figure 1 depicts the year-wise distribution of student-authored publications from 10 September 2008 to 13 December 2014. The number of publications remained unchanged during 2010 and 2011 (n = 2/73; 2.7 %). However, there was a markedly steady increase in the number of publications over the 6-year interval from 1 publication in 2009 to 35 publications by the end of 2014.

**Fig. 2:**
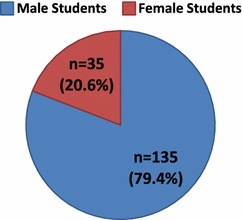
Gender-wise distribution of the medical student authors (n = 170)

**Fig. 3:**
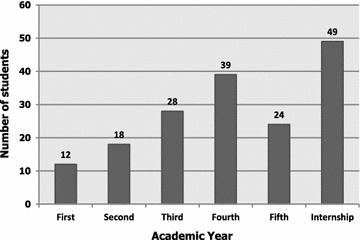
Academic year-wise distribution of the medical student authors (n = 170)

One-hundred and seventy students (n = 170/677; 25.1 %) participated in producing 73 research publications. Among those, 135 (79.4 %) and 35 (20.6 %) students were males and females, respectively (Fig. 2). At the time of research publication, the vast majority of medical student authors (n = 112/170; 65.9 %) were in their clerkship (fourth, fifth and sixth) years (Fig. 3).

Table 1 shows descriptive analysis of multiple variables of the student-authored research publications. More than two-thirds of the publications (n = 50/73; 68.5 %) were first-authored by medical students, as follows: 46 males (63.0 %) and 4 females (5.5 %). Students were corresponding authors in 37 (50.7 %) of the student-authored research publications, all of which were males. The top three explored research areas were clinical science (n = 32/73; 43.8 %), basic science (n = 17/73; 23.3 %) and medical education (n = 16/73; 21.9 %). The top three research types were case reports (n = 30/73; 41.1 %), research articles (n = 24/73; 32.9 %), and letters to editors (n = 11/73; 15.1 %). Fifty-seven (78.1 %) and sixteen (21.9 %) of student-authored research publications took place in local and abroad research institutes, respectively. There were several publications which were co-authored by at least one same student. Interestingly, one outstanding student has contributed (single-authorship and co-authorship) to 36 publications out of the grant total 73 publications (49.3 %).

**Table 1:** Descriptive analysis of multiple variables of the student-authored research publications

Parameter	N (%)
First authors	
Male	46 (63.0)
Female	4 (5.5)
Total	50 (68.5)
Second authors	
Male	19 (26.0)
Female	10 (13.7)
Total	29 (39.7)
Corresponding authors	
Male	37 (50.7)
Female	0 (0)
Total	37 (50.7)
Research area	
Clinical science	32 (43.8)
Basic science	17 (23.3)
Medical education	16 (21.9)
Epidemiology/public health	8 (11.0)
Research type	
Case reports	30 (41.1)
Research articles	24 (32.9)
Letters to editors	11 (15.1)
Review articles	3 (4.1)
Short communications	2 (2.7)
Others	3 (4.1)
Origin of Primary Research Institute	
Local	57 (78.1)
Abroad	16 (21.9)

**Fig. 4:**
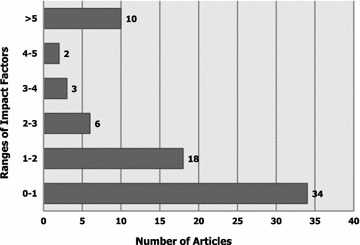
Impact factor-wise distribution of the student-authored research publications (n = 73)

Figure 4 demonstrates the impact factor-wise distribution of student-authored publications. Fifty-two publications (71.2 %) had impact factors ranging from 0 to 2; such publications were mostly case reports. Ten publications (n = 10/73; 13.7 %) had impact factors above 5. Out of those, 3 articles were published in the Lancet (impact factor: 39.2), and 1 article was published in the New England Journal of Medicine [NEJM] (impact factor: 54.4)—all first, second and corresponding authors were medical students. “Esophageal Duplication Cyst”, a brief case report of an intriguingly rare presentation published in NEJM, the world’s leading biomedical journal, was indeed one of the major student-related research achievements at Alfaisal University (Abu-Zaid and Azzam, 2014).

**Fig. 5:**
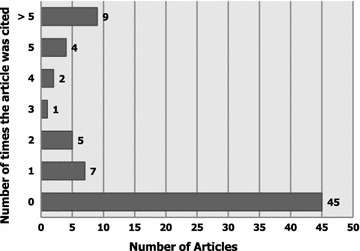
Article citation-wise distribution of the student-authored research publications (n = 73)

Figure 5 exhibits the article citation-wise distribution of the student-authored research publications. Forty-five publications (61.6 %) had not been previously cited, whereas 28 publications (38.4 %) were cited at least once. Interestingly, 9 papers (12.3 %) were cited more than 5 times. The mean ± standard deviation for article citation was 1.9 ± 4.1.

## Discussion

To the best of our knowledge, this is the first ever reported study that quantitatively and qualitatively evaluated the student-authored research publications from an undergraduate medical college in Saudi Arabia in particular, as well as from a developing country in general.

In several countries across the globe, research experience as a medical student has evolved as a mandatory component of the medical graduation requirements (Wickramasinghe et al. 2013). In fact, medical schools in Germany obligate their students to get engaged in full-time research projects prior to graduation and earning the title of “doctor”. In our college—as in the USA and UK institutes (Cursiefen and Altunbas 1998)—engagement in scholarly research activities, and most importantly publishing scientific reports is voluntary although highly encouraged and supported.

There is a directly proportional relationship between senior academic years and higher rate of engagement in undergraduate research activities (Khan et al. 2006). A multi-institutional Canadian study by Siemens et al. (2010) on the attitudes of medical students towards research showed that a greater number of senior fourth-year students were engaged in research compared to their junior second-year counterparts. This finding could be attributed to efficient cross-linking between clinical knowledge and research findings, better awareness of research significance, more attainment of research-specific skills over years, greater time allowance and exposure to research mentors by fourth-year students during their clerkship clinical years, which consequently permitted them to self-assuredly pursue research and publish scientific reports (Khan et al. 2006; Siemens et al. 2010). Likewise, similar results were yielded in our study where senior clerkship medical students had the highest number of publications (Fig. 3).

The trend of higher male participation in research compared to female students has been documented elsewhere in several studies (Burgoyne et al. 2010; Remes et al. 2000; Salgueira et al. 2012; Shahab et al. 2013). A study by Burgoyne et al. (2010) reports that male students feel significantly more confident about transferable and research-specific skills when contrasted to female students. Additionally, female students appear to be less interested in research and tend to prefer devoting more time to academia (curricular activities) than to research (extra-curricular activities) (Abu-Zaid and Alnajjar 2014; Abu-Zaid and Altinawi 2014; Salgueira et al. 2012). Logically, the more time and efforts spent in conducting scholarly research activities, the higher the probability to produce scientific publications (Abu-Zaid 2014). Moreover, we also believe that this gender-biased disparity concerning the number of student-authored publications by males and females may be because of the fact that Alfaisal University—College of Medicine began enrolling male students in Fall 2008 and female students in Fall 2011. Therefore, male students have had the advantage of three surplus years to produce publications.

Globally, the most common fields of medical student research publications (sequentially ordered) include: psychiatry, general medicine, medical education, oncology and community medicine (Wickramasinghe et al. 2013). In our study, the number of clinical science publications was roughly 2 times the number of basic science publications. A study by Druss and Marcus (2005) reports that there has been a movement from basic science to clinical science research over the course of the past two decades. Moreover, similar reports were also made in a different study, which underscored minimal (9 %) basic science research involvement compared to the increased affinity for clinical science related research such as retrospective chart reviews and case reports (Siemens et al. 2010). In our study, one plausible reason for the high number of publications in clinical science (particularly surgical oncology) could be related to the tertiary healthcare nature of the students’ clerkship training institute; the vast majority of patient cases are oncology-related referrals from the other local hospitals across the country.

Wickramasinghe et al. (2013) in their research article titled: “patterns and trends of medical student research”, review articles, cross-sectional studies and case reports were among the commonest types of student-authored publications listed under PubMed^®^ and Scopus^®^ search engines. Similar results were echoed in our study.

One of the strengths of this study is that to the best of our knowledge, this is the first ever report which attempted to descriptively analyze medical student-authored publications from Saudi Arabia, specifically, and developing countries, generally. Furthermore, our study intends to encourage other medical colleges in Saudi Arabia to follow our footsteps and present their analyses of student-authored research publications from their institutions. This will be very valuable in providing a more valid generalized picture on the status of student-authored publications in Saudi Arabia and to look for similarities and/or differences across institutes. Afterwards, barriers to scientific publishing can be better explored.

One major limitation of this study is that student-authored book chapters, and manuscripts currently under-review (submitted) or published in non-PubMed-indexed journals were not included in the study. This may have affected the overall analysis of all scientific outputs produced by the medical students at our College.

## Conclusion

Students demonstrated positive attitudes towards publishing and significantly contributed to the institution’s pool of research publications. An improvement to this study would be to extensively assess how students’ demographics (for example, gender and academic year), personal traits, cumulative grade point average (cGPA), future medical specialty, and previous research experiences influence the publishing practices of medical students. Future studies include: Alfaisal University—College of Medicine students’ perceived attitudes and barriers towards oral/poster presentations in scientific meetings.
